# Quarterly Retrospect

**Published:** 1861-04

**Authors:** 


### *Quarterly Retrospect.*

*April*, 1861.

THE last Quarterly Return of the Registrar-General (February, 1861), containing summaries of the marriages registered in the third, and of the births and deaths in the fourth, quarter of 1860, contains also a brief account of the VITAL CREDIT AND DEBIT for that year. If we had had, without the aid of statistics, to form our notions of the healthiness or unhealthiness, and the prosperity or not of the kingdom in 1860, our conclusions would have been upon the whole somewhat gloomy. The general intemperateness of the seasons, the backwardness or imperfectness of several of the most important crops, the dearness of food, the disturbances in the labour market, and the sufferings in several localities arising from this source, altogether would have formed a sad and depressing picture, from which little consolation could have been obtained. When, however, we turn from the vague ideas formed from general observation to the precise data afforded by figures, the silver lining of the dark cloud is at once turned to the light.

The *births* of 683,430 children were registered in 1860, and the annual birth-rate was 3·418, being ·014 above the annual average. The daily births, taking one day with another throughout the year, were 1867. The *natural increase* of the population of England and Wales amounted to 260,930 souls, averaging 713 daily. If the natural increase in Scotland and Ireland was at the same rate, the population of the United Kingdom must have augmented at the rate of 1069 souls each day. The *deaths* were 422,500, and the annual rate of mortality 2113, or a little more than 21 in 1000, the average of the preceding ten years being 22. Thus one life in every 1000 was saved.

Notwithstanding, however, the favourable vital balance shown by these figures, this is far from being what it ought to be. There is still a large amount of life-waste. If, for example, the mortality of the whole of England and Wales, in the last quarter of 1860, had been at the rate only of that experienced in the healthiest districts, the deaths, instead of being 102,557, would have amounted solely to 79,283. Thus, it may be estimated that during the 92 days of the quarter, 23,274 persons "died unnatural deaths in the least unhealthy country in Europe.

"An excess of deaths," writes the Registrar-General, in his Return for the

Second Quarter of 1860, "which is not decreed by inexorable fate, may very properly be termed 'unnatural,' though it is quite true that, only the conditions being different, it is nature that *killeth* as well as *giveth life.*

"It is a remarkable and interesting fact, that if 2,000,000 of acres on which the chief towns of England are situated, be distinguished from the remaining 34,000,000 that hold small towns, and country parishes, it is found that the rate of mortality on the former (2·305 per cent. per annum) was *below* the average. The average rates were respectively 3·346 and 2·028. Although the time may be distant when cities will be as healthful as rural districts, or the inferiority which our English poet ascribed to 'the town,' as the handiwork of man, become much less apparent in point of salubrity than it is at present, it cannot be questioned that large populations have even now advantages of a nature favourable to health which villages do not possess. The highest attainable health is probably to be sought in a happy combination of both states—*rus in urbe.* The words of an excellent popular writer may prove to be no dream, but a well-founded expectation; he believes that we shall ultimately obtain 'a complete interpenetration of city and country, a complete fusion of their different modes of life, and a combination of the advantages of both, such as no country in the world has ever seen.'* And it may be asked, whether it is forbidden by this last expression to accept as a perfect model even Nebuchadnezzar's Babylon, which the distinguished writer himself has extolled."

A curious and very instructive illustration of the influence of the less apparent or less heeded sanitary laws upon mortality is afforded by the report of the Registrar of Holy Trinity, Coventry. Nothing in the course of last year excited such painful interest and so great sympathy as the distress among the ribbon-weavers of that city. The gentleman referred to states that notwithstanding the unprecedented suffering which had prevailed there in consequence of the prostration of the ribbon-trade, the rate of mortality was extremely low, "there being only 67 deaths against 132 deaths in the corresponding quarter of last year; 98 in 1858, and 100 in 1857." Upon this the Registrar-General remarks: "The care of the mothers of Coventry has, it would seem, counteracted some of the effects of privation; so that neglect of their homes by mothers at work in the manufactories, is apparently more fatal than starvation." To this observation we may very fittingly append another from the first Quarterly Return for 1860. In commenting upon the ungenial nature of the season in the first quarter of the year, the Registrar-General remarks:—

"It is evident that the severity of the season must have been felt all over the kingdom; yet, notwithstanding the effects of the weather, the mortality decreased wherever sanitary arrangements were sedulously worked. Even careful observers, sometimes, it may be remarked, refer the excessive mortality of their districts to the weather, or to some other cause over which people have little or no control. They do not bear in mind that foul air is more fatal

* Charles Kingsley's *Miscellanies:* "Great Cities."

than cold air, and that food, and clothing, and active employment keep out the cold."

It is pleasant to learn, from the same authority, previous to entering upon the second subject of our Retrospect, the displacement of population in the metropolis and large towns, that notwithstanding the insanitary state of town districts, the effects of sanitary improvements within these are beginning to tell plainly upon the mortality. Thus, "In the population of town districts exceeding 8,000,000 at the last census, the mortality was at the rate of 23 in 1000; in the country districts of more than 9,000,000, the rate of mortality was 18 in 1000; so the chances of dying in the two groups of districts were as 23 to 18." But now "the mortality of the town districts has declined from 25 to 23, and of the country districts from 19 to 18."*

The questions involved in the displacement of population in the metropolis and large towns, by the extension of railways into them, or by those internal changes commonly summed up under the term "improvements," are very complex. Even the sanitary question is susceptible of a twofold application; for, on the one hand, the clearing away of old and dilapidated blocks of buildings, and the substitution of more open areas for close alleys and confined courts, are sanitary advantages of the very highest order; while, on the other hand, the overcrowding which may be occasioned elsewhere by the displacement of population, is a disadvantage of a very serious character. That this disadvantage has been brought about in the metropolis by the structural and roadway changes which have taken place in the City from time to time, would appear to be certain from the statistical records of the distribution of the population at different periods. Thus in a recent debate in the House of Lords (March 11) upon this subject, and in the course of which the Earl of Derby moved that—

"The Select Committees of the Metropolitan Railways do inquire into and report upon the number of houses, and of inhabitants, likely to be removed by the works of the respective railways; and whether any provision has been made, or is required to be made, for diminishing the evils consequent on a large simultaneous displacement of the labouring population,"

That nobleman said:—

"I have had placed in my hands some statistics—with the details of which I shall not trouble your lordships now, as they will be printed in a few days—which show the comparative populations and number of houses in the City parishes, both within and without the walls, in the years 1801 and 1851. Your lordships will not, perhaps, be surprised to hear that the population of the City parishes was slightly smaller in 1851 than it was in 1801, there being a diminution of some 200 or 300 upon a total of 129,000 or 130,000. But it is remarkable that while the population had remained stationary, the number of houses

* Quarterly Return, February 1, 1861, p. 4.

since 1801 had not only not increased, but had actually diminished to the extent of about 3000, and therefore it appears that the same population which in 1801 inhabited 17,000 houses, were in 1851 crowded into 14,000 houses. There is something, too, which is rather curious when we look at the respective districts. In the parishes within the walls there has been the greatest decrease in the number of houses—2776; but then there has been a corresponding diminution of population in those parishes to the extent of 19,000 souls. The result is that the average number of inhabitants of each house within the walls is the same in 1851 as it was in 1801—viz., 7½ to each house; but in the City parishes without the walls, to which the poor have been driven by improvements effected in the metropolis, I find that the houses have decreased in number about 300, while the population has increased by 19,000. Thus, while the proportion of inhabitants to each house in the inner parishes is 7½, in the outer parishes it has increased to 9 and 6-7ths in each house. The figures which I have now quoted have reference to the year 1851, but your lordships will be perfectly aware that since then great improvements—and great improvements they undoubtedly are—have been going on in the City, the result of which has been to displace a very large number of the inhabitants. In a pamphlet published by Mr. Pearson, the City Solicitor, referring to the immense improvements which have taken place in Cannon-street, Victoria-street, and elsewhere, it is stated:—'The noble street improvements undertaken by the Corporation have swept, and are about to sweep away thousands of industrious artisans and mechanics from their humble dwellings, to make way for the spacious streets and splendid warehouses destined in this age of progress to take their place. To supply the lack of dwellings which these clearances make, the Corporation have recently voted a large sum to purchase land and erect lodging-houses in the neighbourhood of Victoria-street.'"

The latter intention, however, has not been fulfilled.

In the course of the debate Lord Granville said, with particular reference to the extension of metropolitan railways, that it appeared to him there were only three remedies for the evils complained of: one was to force the railway companies to build a corresponding number of houses in another part of the town. Another was to organize cheap trains to carry artisans from healthy dwellings in the suburbs to the scene of their work. This, indeed, is already being carried out by agreement with the City authorities, three of the largest companies having undertaken to convey one thousand passengers per day from a distance not exceeding ten miles to London and back for twopence each. A third was, to suspend "these great works of metropolitan improvements."

Commenting upon Lord Derby's speech and the questions raised by it, the *Times* observed that—

"In nineteen cases out of twenty it is better a person—be it man, woman, girl, or boy—should live a good mile from his work. Of artisans' and labourers' villages we would wish to speak diffidently, though we see no reason —except habit and prejudice—why ten or twenty thousand workpeople should not spend the night in the country as well as their masters. But, putting this out of the question, or regarding it simply as an experiment waiting for proof, we do think that Lord Derby and his friends are wasting their philanthropy on workpeople wishing to live within a quarter of a mile of their work. If the working people of the City are compelled to find room at a greater distance from their work, there will be builders and speculators ready to supply their wants. This is not an affair for railway companies. The Corporation of the City has tried it and failed. It requires more personal attention than any large body of men can give to it. Moreover, when any one of the great West-end proprietors lays out a hundred acres in squares and streets, he does not concern himself with the working people. They must shift as they can, and go as far off as possible. In a word, it cannot be done by law. All that is wanted is that the rules of public health should be put in force, and no lodging-houses or other dwellings allowed to be built that shall be liable to breed a pestilence. This we all of us have a right to demand, but it is a matter of dimensions, drainage, ventilation, and other internal arrangements, and does not affect the locality of the dwelling. Government, therefore, has nothing to do with providing dwellings for the poor, and has no more right to impose an obligation of this sort on railways, than on anybody who pulls down a dwelling-house to build something else—a church, for example—in its place. The interference is both idle and contrary to the usages of this country. It can end in no good. We accept railways with their consequences, and we don't think the worse of them for ventilating the City of London.—(March 12.)

These remarks of the *Times* are undoubtedly right in the main. But while accepting railways or any other metropolitan improvements, or those of any other city, it is well that the immediate social discomfort and evils they may give rise to should not be allowed to be huddled away under the easy-going proposition that "it will all come right in the end." This the debate in the House of Lords, and the instructions which it led to, are well calculated to bring about. That the evils arising from the displacement of population, by the destruction of houses in those portions of the metropolis traversed or about to be traversed by railways, or opened out by other improvements, are at all comparable in degree with those which would arise from the same localities remaining *in statu quo*, we cannot believe. As a rule, the railways, governed in no small degree by economical considerations, penetrate through the most wretched districts—districts which are centres of all those evil influences which act most powerfully in the physical and social degeneration of the people. It needs but little experience of the inhabitants of these localities to know that they cannot be tempted to move spontaneously from them. Nay, even if improved buildings for their accommodation are erected in their midst, it is not always easy to tempt them to occupy those buildings; while the huge objection remains that the new home is, with the exception of its internal arrangements, exposed to the same pestilent insanitary evils as the old one. Now, the only method of obtaining that change which is desirable for the sanitary and social welfare of the locality must arise from such alterations as will sweep away its old structures, or at least open them out more freely to the heavens, or completely disperse or largely diminish its population. This is the great good effected by "improvements" which largely affect the houses in the more closely and older built portions of great towns and cities. If, of course, the evils thus got rid of, or greatly relieved in one place, were immediately reproduced in an equal or exaggerated form in another, this unhappy consequence would more than counterbalance the good. But we see no reason to apprehend that this will ever be (and we doubt if it has ever been) the case. The displacement of population is generally slowly effected, and as a rule the dispersion is great; again, the changes giving rise to the displacement have always gone on contemporaneously with a steadily increasing and more perfect general and local care for the public health, so that every displacement has led to a resultant in favour of the health and social status of the community. In short, those improvements which lead to the destruction of houses in crowded localities, do for the sanitary conditions of cities and towns what else could only be obtained by an arbitrary exercise of power. They give the occasion for a better and fuller exercise of the great laws of public health; they furnish the most effective means for the internal sanitary improvement of the mass of our town populations; and it is evident that the immediate evils which they give rise to will be met by those means which most efficiently secure the carrying out of ordinary sanitary regulations in all localities without distinction. It is also evident that the new house accommodation that is required to anticipate or relieve the overcrowding induced by displacement of population should be, if sanitary regulations are to have any weight, upon the outskirts of towns. That the legislature may interfere with good to secure a right attention to sanitary requirements both in the structure and management of new buildings for the masses is probable, but that it could interfere beneficially to *cause* such buildings to be erected is very problematical.

*Apropos* of Metropolitan improvements, it is pleasant to find, from certain observations made in the House of Commons on the 2nd of March, that the project of embanking the Thames is not to be suffered to fall into the shade. We may perhaps indulge a hope that that great work may be commenced, if not completed, in our own time; and we may venture to look forward, without drawing too much upon the imagination, to a period when both banks of the river above Somerset House will present broad and pleasant walks in which the pedestrian may delectate. For it is to be presumed that the embankment being carried out, buildings would not again be permitted to touch the water's edge; and that, with the annihilation of the mud banks and the increased scour of the stream, the offensiveness of the river would be so greatly diminished (if not in the end, particularly when the new system of drainage comes into full operation, got rid of) that the swans would once more be tempted to flock down to the vicinity of the bridges, and citizens would find the water side an exhilarating and healthful resort for themselves and families.

The chief matter of moment during the past quarter, in connexion with insanity, was the second reading of the Lunacy Regulation Bill in the House of Lords (*March* 18th). We quote, without comment, the observations made at the time by the Lord Chancellor and the Earl of Shaftesbury.

"The LORD CHANCELLOR, in moving the second reading of this Bill, said a great deal had been done to improve the position of lunatics by Lord Brougham, Lord Lyndhurst, and Lord St. Leonards; but a great deal remains to be accomplished. There were many persons of unsound mind possessing a small amount of property, which a commission of lunacy would wholly absorb. But unless there was a commission, the property could not be made available for the benefit of the owner. It was proposed by the Bill that, if it were made out to the satisfaction of the Lord Chancellor that these persons, who had incomes under a fixed sum, were lunatic, and if, after notice, they made no objection, the Lord Chancellor should have the power to dispose of the property as if a commission had issued and they had been regularly found insane. He admitted that it was a very stringent measure, and one which he should not propose if it were not absolutely necessary. Instances showing its necessity had been furnished by the commissioners. In 1858 a governess, at that time lunatic, became entitled to about 100*l*., and it continued to this day to be held by a joint-stock bank, who refused to pay it over without the authority of the Lord Chancellor. The Lord Chancellor could give no authority, unless a commission found her insane, and the expense would wholly absorb the property. In September, 1858, A. B., then in a country asylum, became entitled to a legacy of 200*l*. His own funds were exhausted, and the trustees refused to apply the legacy for his benefit, because they could receive no proper discharge. There was a patient in St. Luke's Hospital possessed of 100*l*. in a savings-bank. His wife could not maintain him. She could scarcely maintain herself. She had repeatedly requested the commissioners to appropriate the money to his use, but they had no power to do so. There were other cases to the same effect, and he hoped that if the Lord Chancellor for the time being were intrusted with the power proposed, it would be always exercised for the benefit of the unhappy lunatics. (Hear, hear.) By the present law, a person of whose lunacy there was not the smallest doubt could insist upon a second inquiry. Some years ago, a Mrs. Cuming, after an inquiry which lasted sixteen days, was found lunatic on very satisfactory evidence. She insisted on another inquiry. Lord St. Leonards saw her, and tried to dissuade her; but, whether from her want of understanding or from her being prompted by others who might have had mercenary motives, she persisted, and another inquiry would have taken place had not Providence interfered and cut her life short. It was proposed by the Bill to give the Lord Chancellor a discretion to grant or refuse a second inquiry, after having seen the lunatic and taken the best means in his power to come to a right conclusion. It was also proposed to give power to grant retiring allowances to the visitors to the extent of two-thirds of their salary. Two of the present visitors had been in office about thirty years. He believed they had done their best to discharge the duties imposed on them; but they were now of a very advanced age and physically unfitted to continue in office. It had been proposed that the Chancery visitors should be abolished, and that lunatics under the charge of the Lord Chancellor should be visited by the visitors of the General Lunacy Board. He was afraid, however, that this proposition could not be carried out. The

General Board had already more to do than they could well get through, and if there were any increase to their numbers it must be at the public expense; whereas the Chancery visitors did not cost the public anything. It had been proposed, too, in order that the General Board might take charge of them, that the Chancery lunatics should be brought together, in the neighbourhood of some central railway station; but this was absolutely impracticable. They were scattered about the country in their own houses, among their friends, or in the charge of clergymen, physicians, and so on, so that it was impossible to bring them all together. It was most essential, too, that there should be direct communication between the lunatics and the Court, and this could only be done by releasing the Chancery visitors. The only other provisions of the Bill were one for declaring that Masters in Lunacy should not be able to sit in the House of Commons, about which a doubt had been raised a short time ago; and another to make the Registrar in Lunacy a permanent officer. The noble and learned lord concluded by moving the second reading of the Bill.

"The Earl of SHAFTESBURY heartily concurred in the principle and details of the Bill, which he believed to be calculated to promote the welfare of this unhappy class of persons. The cases mentioned by the noble and learned lord were merely representative cases. Many others equally striking might have been brought forward. It was not the General Lunacy Commissioners who had proposed that the Chancery lunatics should be transferred to them, for they had already a great deal more work than they could do; but it was the wish and desire of the House of Commons, as the system of Chancery inspection was so imperfect and infrequent, that the visitation should be transferred to the General Board. The Board said they would be perfectly ready, provided certain facilities were given to them. If the noble and learned lord desired to retain the visitation in the hands of the Court, he hoped that the visitors would be required to devote themselves exclusively to the work. It would not do for them to devote one-half of their time to visiting, and another part to the general duties of their profession. Their whole time, strength, and attention must be given to the visitation, otherwise the system would not attain that pitch of efficiency which the noble and learned lord desired."

The Bill was then read a second time.

